# Phospholipase C-β3 is dispensable for vascular constriction but indispensable for vascular hyperplasia

**DOI:** 10.1038/s12276-024-01271-6

**Published:** 2024-07-01

**Authors:** Seo Yeon Jin, Jung Min Ha, Hye Jin Kum, Ji Soo Ma, Hong Koo Ha, Sang Heon Song, Yong Ryoul Yang, Ho Lee, Yoon Soo Bae, Masahiro Yamamoto, Pann-Ghill Suh, Sun Sik Bae

**Affiliations:** 1 Medical Research Institute, Department of Pharmacology, Pusan National University School of Medicine, Yangsan, Republic of Korea; 2 Department of Immunoparasitology, Osaka University, Suita, Japan; 3 Department of Urology, Pusan National University Hospital, Busan, Republic of Korea; 4 Department of Internal Medicine, Pusan National University Hospital, Busan, Republic of Korea; 5 Aging Research Center, Korea Research Institute of Bioscience and Biotechnology, Daejeon, Republic of Korea; 6 Carcinogenesis and Metastasis Research Branch, National Cancer Center, Goyang, Republic of Korea; 7 Department of Life Science, Ewha Womans University, Seoul, Republic of Korea; 8 Korea Brain Research Institute, Daegu, Republic of Korea

**Keywords:** Phosphoinositol signalling, Carotid artery disease

## Abstract

Angiotensin II (AngII) induces the contraction and proliferation of vascular smooth muscle cells (VSMCs). AngII activates phospholipase C-β (PLC-β), thereby inducing Ca^2+^ mobilization as well as the production of reactive oxygen species (ROS). Since contraction is a unique property of contractile VSMCs, signaling cascades related to the proliferation of VSMCs may differ. However, the specific molecular mechanism that controls the contraction or proliferation of VSMCs remains unclear. AngII-induced ROS production, migration, and proliferation were suppressed by inhibiting PLC-β3, inositol trisphosphate (IP_3_) receptor, and NOX or by silencing PLC-β3 or NOX1 but not by NOX4. However, pharmacological inhibition or silencing of PLC-β3 or NOX did not affect AngII-induced VSMC contraction. Furthermore, the AngII-dependent constriction of mesenteric arteries isolated from PLC-β3^∆SMC^, NOX1^−/−^, NOX4^−/−^ and normal control mice was similar. AngII-induced VSMC contraction and mesenteric artery constriction were blocked by inhibiting the L-type calcium channel Rho-associated kinase 2 (ROCK2) or myosin light chain kinase (MLCK). The activation of ROCK2 and MLCK was significantly induced in PLC-β3^∆SMC^ mice, whereas the depletion of Ca^2+^ in the extracellular medium suppressed the AngII-induced activation of ROCK2, MLCK, and vasoconstriction. AngII-induced hypertension was significantly induced in NOX1^−/−^ and PLC-β3^∆SMC^ mice, whereas LCCA ligation-induced neointima formation was significantly suppressed in NOX1^−/−^ and PLC-β3^∆SMC^ mice. These results suggest that PLC-β3 is essential for vascular hyperplasia through NOX1-mediated ROS production but is nonessential for vascular constriction or blood pressure regulation.

## ROCK2 activation: linking angiotensin II to hypertension

Angiotensin II is important in heart health. It makes blood vessels tighten and grow. This study looked at how AngII affects the creation of reactive oxygen species (ROS, molecules that change cell function) in vascular smooth muscle cells (VSMCs, cells in blood vessel walls). The researchers tested how stopping certain cell signals changes ROS creation and cell behaviors like growth and movement. They found that a specific protein, PLC-β3, and an enzyme, NOX1, are key in this process. Stopping these molecules could lower ROS levels and change cell growth and movement, important for blood vessel health. Interestingly, these molecules didn’t affect blood vessel tightening, also controlled by AngII. This study could help develop new treatments for blood vessel diseases, potentially helping manage conditions like high blood pressure and heart disease.

This summary was initially drafted using artificial intelligence, then revised and fact-checked by the author.

## Introduction

Angiotensin II (AngII) maintains systemic blood pressure through various mechanisms within the cardiovascular and renal system^1^ and has been implicated in several cardiovascular diseases, including hypertension, heart failure, and atherosclerosis, and mechanistically in vascular smooth muscle cell (VSMC) contraction and vascular remodeling through the induction of hyperplasia.

Several AngII receptors have been identified, and the AT_1_ receptor, which is expressed in VSMCs, has been extensively studied^2^. The AT_1_ receptor is coupled to G_q/11_ or G_12/13_, which have phospholipase C (PLC) that acts as an effector protein. Following G_q/11_ activation, second messengers such as inositol trisphosphate (IP_3_) and diacylglycerol (DAG) are generated by PLC activation. IP_3_ stimulates calcium release from the endoplasmic reticulum and thus increases classical protein kinase C (PKC) activation. The calcium binding protein calmodulin is a primary target of elevated intracellular calcium, and the Ca^2+^-calmodulin complex binds to myosin light chain kinase (MLCK), which culminates in MCL phosphorylation and VSMC contraction^3^. However, intracellular calcium could be elevated by additional mechanisms, such as the depolarization of Cav1.2 and Cav1.3 (L-type calcium channels (LTCCs)), which are the major causes of calcium elevation in arterial smooth muscle^4,5^. The contractile status is further maintained by Rho-associated kinase (ROCK)-mediated phosphorylation and inactivation of myosin light chain phosphatase (MLCP)^6^. In addition, AngII induces vascular hyperplasia through the activation of receptor tyrosine kinases (RTKs)^7^. However, the mechanisms underlying AngII-dependent proliferation and migration have not been elucidated.

The 13 mammalian PLC isozymes identified can be divided into 6 subgroups: PLC-β, PLC-γ, PLC-δ, PLC-ε, PLC-ζ, and PLC-η^8^. PLC-γ is activated by RTKs, whereas PLC-β is activated by G protein-coupled receptors (GPCRs). Intracellular calcium mobilization stimulates PLC-δ and PLC-η, and Rho stimulates PLC-ε, but the mechanism responsible for PLC-ζ stimulation is unknown. PLC-β has four different subtypes (PLC-β1-4), and differential expression patterns and functions of these subtypes have been reported. Although the signaling cascades from AngII-dependent PLC-β activation to calcium mobilization in VSMCs are well defined, direct evidence regarding the regulation of contraction, proliferation, and migration of VSMCs is lacking.

AngII stimulates vascular contraction and hypertrophic growth through multiple signaling pathways that involve the production of reactive oxygen species (ROS)^9^. Multiple sources generate ROS, such as nicotinamide adenine dinucleotide phosphate (NADPH) oxidase (NOX), xanthine oxidase, and the mitochondrial respiratory chain^10^. NOX actively produces ROS upon exposure to extracellular stimuli, and the NOX family consists of seven isoforms, which include NOX1-5 and dual oxidase (DUOX) 1 and 2^11^. NOX1 is fully activated by interacting with NOXO1, p47^*phox*^, and p22^*phox*^, whereas NOX2 is activated by binding with p47^*phox*^, p67^*phox*^, p22^*phox*^, and Rac1^12,13^. NOX5 is activated by intracellular calcium binding, whereas NOX4 is activated by binding with POLDIP2 and p22^*phox*^^14,15^. Furthermore, NOX1 and NOX4 are major sources of ROS in VSMCs^16^.

Several reports have implicated PLC in the AngII-induced production of ROS. For example, PLC-mediated PKC activation is involved in the production of ROS during AngII stimulation^17^. Additionally, the AngII-induced activation of PLC generates IP_3_ and DAG, and subsequent activation of PKC facilitates the activation of the NOX complex to produce ROS^18^. Furthermore, AngII regulates several VSMC physiological processes, such as contraction, proliferation, and migration.

In the present study, we describe the involvement of a specific isoform of PLC-β and NOX in the regulation of ROS generation and their functional roles in the modulation of VSMC physiology.

## Materials and methods

### Materials

Dulbecco’s modified Eagle’s medium (DMEM), fetal bovine serum (FBS), trypsin-ethylenediaminetetraacetic acid (trypsin-EDTA), and penicillin/streptomycin (antibiotics) were purchased from HyClone Laboratories Inc. (Logan, UT, USA). Anti-PLC-β1, anti-PLC-β2, anti-PLC-β3 and anti-PLC-β4 were obtained from Santa Cruz (California City, CA, USA). The anti-actin antibody was purchased from MP Biomedicals (Aurora, OH, USA). Anti-SMA, anti-calponin, 2’,7’-dichlorofluorescein diacetate (DCF-DA), PLC inhibitor (U73122, U73343), PKC inhibitor (GF109203X, rottlerin), NADPH oxidase inhibitor (apocynin, DPI), ROS scavenger (N-acetylcysteine), L-type calcium channel inhibitor (nifedipine), Rho-kinase inhibitor (Y27632), angiotensin II (AngII), and IP_3_ receptor antagonist 2-aminoethoxydiphenyl borate (2-APB) were obtained from Sigma-Aldrich (St. Louis, MO, USA). The anti-SM22α antibody was obtained from Abcam (Cambridge, UK). Peroxy Orange 1 (PO-1) was purchased from R&D Systems (Minneapolis, MN, USA). Dihydroethidium (DHE), 4’,6-diamidino-2-phenylindole (DAPI), Alexa Fluor 488-conjugated goat anti-mouse secondary antibody and Cy-3-conjugated goat anti-rabbit secondary antibody were purchased from Molecular Probes, Inc. (Carlsbad, CA, USA). IRDye700- and IRDye800-conjugated rabbit/mouse secondary antibodies were obtained from Li-COR Bioscience (Lincoln, NE, USA). ChemoTx membranes (8 μm pore size) were obtained from Neuro Probe, Inc. (Gaithersburg, MD, USA). The IP_3_ receptor antagonist 2-aminoethoxydiphenyl borate (2-APB) and all other chemicals were purchased from Sigma-Aldrich (St. Louis, MO, USA) unless otherwise indicated.

### Cell culture and transfection

To isolate VSMCs, Sprague‒Dawley rats (3 weeks old) were euthanized by intraperitoneal injection of sodium pentobarbital (60 mg/kg) via tissue explants. Thoracic aortas were isolated, and surrounding fat and connective tissues were discarded. The vessels were cut longitudinally, and the lumens were scraped with a razor blade to remove the intima. The vessels were then cut into 3–5 mm long pieces, and the lumen was explanted side down on collagen-coated culture dishes. Seven days later, the tissue fragments were discarded, and the sprouting VSMCs were collected (referred to as P0 (passage 0)). Synthetic VSMCs were cultured at low density (<20%). To induce the phenotypic conversion of VSMCs, synthetic VSMCs (P0) were cultured at high density (~100%). Cells cultured between P2 and P5 were defined as contractile-type VSMCs.

### Western blotting

Cells were lysed in 20 mM Tris-HCl, pH 7.4, 1 mM EGTA/EDTA, 1% Triton X-100, 1 mM Na_3_VO_4_, 10% glycerol, 1 μg/ml leupeptin and 1 μg/ml aprotinin. After centrifugation at 12,000 rpm, 30 μg of total protein was loaded onto 10% polyacrylamide gels and transferred onto nitrocellulose membranes. The membranes were incubated with the indicated primary antibodies and IRDye-conjugated secondary antibodies, and the protein bands were visualized using an infrared image analyzer (Li-COR Bioscience).

### Migration assay

VSMCs were serum-starved for 12 h, plated on a ChemoTx membrane, detached with trypsin-EDTA, and washed with serum-free DMEM. For the migration assay, the lower surface of a ChemoTx membrane was coated with type I collagen for 30 min, and 1 × 10^5^ serum-starved cells in a 50 μl volume were placed on the upper surface. Migration was induced by submerging the ChemoTx membrane in serum-free DMEM for 6 h, after which the membrane was fixed with 4% paraformaldehyde, and nonmigrated cells on the upper surface were removed with a cotton swab. The membrane was stained with DAPI, and the migrated cells were counted using a fluorescence microscope at ×10 magnification (Axiovert200, Carl Zeiss, Jena, Germany).

### Cell proliferation assay

VSMCs (1 × 10^5^) were plated on a 6-well plate and stimulated with AngII (1 μM) for 2, 4, or 6 days in culture medium, after which the cells were fixed with 4% paraformaldehyde. Nuclei were stained with DAPI and examined under a fluorescence microscope at ×20 magnification.

### ROS, H_2_O_2_, and O_2_^−^ generation assay

VSMCs were cultured in 48-well plates, serum starved for 12 h, treated with 2’,7’-dichlorofluorescein diacetate (DCF-DA, 20 μM), O_2_^−^ sensitive dye dihydroethidium (DHE, 1 μM) for 1 h, H_2_O_2_ sensitive dye peroxy orange 1 (PO-1, 5 μM) for 30 min, stimulated with AngII (1 μM) for 15 min under the indicated conditions, and washed three times with phosphate-buffered saline (PBS). Fluorescence was detected using a fluorescence microscope at ×10 (Axiovert200, Carl Zeiss, Jena, Germany). Pixel intensities were measured using MetaMorph software (Molecular Devices, Sunnyvale, CA, USA).

### Cytosolic calcium concentrations

Intracellular calcium concentrations were measured using Fura-2 AM (a calcium-sensitive fluorescent dye), as previously described^19^. Briefly, 1 × 10^6^ VSMCs were incubated with 3 μM Fura-2/AM at 37 °C in serum-free DMEM with stirring for 50 min, after which 1 × 10^6^ cells were aliquoted into Locke’s solution (154 mM NaCl, 5.6 mM KCl, 1.2 mM MgCl_2_, 5 mM HEPES pH 7.3, 10 mM glucose, 2.2 mM CaCl_2_, and 0.2 mM EGTA). Fluorescence was measured at an emission wavelength of 500 nm using excitation wavelengths of 340 and 380 nm.

### Collagen gel contraction assay

Confluent VSMCs were subjected to trypsin digestion and resuspended in serum-free DMEM. The cell suspension was then mixed on ice with a collagen gel solution (8 mg/ml of collagen type I in 2X PBS, pH 8.0) to a concentration of 5 × 10^5^ cells/ml and 4 mg/ml of collagen gel in the mixture. Then, 100 μl of this mixture was slowly added to 12-well plates, which were incubated at 37 °C for 30 min to cause polymerization. The gels obtained were then floated in serum-free DMEM for 6 h and treated with AngII to initiate contraction. Images were captured using a digital camera. Collagen gel contraction was defined as a decrease in the gel area, as determined by ImageJ (National Institutes of Health, MD, USA). The results are expressed as relative gel areas, which were calculated by dividing the gel areas at specific time points by the initial gel areas.

### Immunohistochemistry

Aortic tissues were fixed with 4% paraformaldehyde, permeabilized with 0.2% Triton X-100, and incubated with a primary antibody for 3 h and then with Cy3- or Alexa Fluor 488-conjugated secondary antibody for 1 h. Samples were mounted using the anti-fading reagent (2% n-propyl galate in 80% glycerol/PBS solution), and images were obtained using a confocal microscope (F1 Flour, Nanoscope Inc., Daejeon, Korea).

### Short-hairpin RNA and constructs

To silence PLC-β3, NOX1, NOX4 and PKC-δ, shPLC-β3 (5′-CGC GGG AGT AAG TTC ATC AAA-3′), shNOX1 (5′-TCA TAT CAT TGC ACA TCT ATT-3′), shNOX4 (5′-AAA CCG GCA GAG TTT ACC CAG-3′), and shPKC-δ (5′-CGC TGA GTT CTG GCT GGA CCT-3′) oligonucleotides with an *AgeI* site at the 5′-end site and an *EcoRI* site at the 3′ end were designed, and sense and antisense oligonucleotides were synthesized (XENOTECH, Daejeon, Korea). Both complementary oligonucleotides were mixed, heated to 98 °C for 5 min, and cooled to room temperature. The annealed nucleotides were subcloned and inserted into the *AgeI/EcoRI* sites of the pLKO.1 lentiviral vector.

### Lentiviral knockdown

For gene silencing, HEK293-FT packaging cells (Invitrogen, Carlsbad, CA, USA) were cultured to ~70% confluence in 100-mm cell culture dishes and triple transfected with 20 μg of pLKO.1 lentiviral vector containing shPLC-β3, shNOX1, shNOX4, shPKC-δ, 5 μg of Δ8.9, or 5 μg of pVSV-G using the calcium phosphate method. The medium was replaced with fresh medium 8 h posttransfection. Lentiviral supernatants were harvested 24 h or 48 h posttransfection and passed through 0.45-μm filters. Cell-free viral culture supernatants were used to infect contractile VSMCs in the presence of 8 μg/ml polybrene (Sigma). Infected cells were isolated by selection with 10 μg/ml puromycin for 2 days.

### Analysis of mRNA expression

mRNA expression was quantified by reverse transcription PCR (RT‒PCR) analysis of total RNA isolated using TRIzol reagent according to the manufacturer’s instructions (Life Technologies, Grand Island, NY). Total RNA (1 μg) was reverse transcribed into cDNA using the ImProm-II reverse transcription system (Promega), which was then amplified by PCR using specific primers (Supplementary Table 2). Equal amounts of RT‒PCR products were separated on a 2% agarose gel and visualized by ethidium bromide staining (Sigma-Aldrich, St. Louis, MO). The expression levels of NOX1 and NOX4 were quantified by real-time quantitative PCR (Q-PCR) using specific primers (Supplementary Table 3) and analyzed using the comparative *C*_t_ method.

### Animals

Mice lacking PLC-β3 in VSMCs (PLC-β3^∆SMC^) were generated as previously reported^20^. Briefly, a DNA fragment containing exon 2 and exon 13 of murine PLC-β3 was cloned and inserted into the pBluescript phagemid system. The LoxP sequence was inserted at introns located between exons 4 and 5 and exons 8 and 9. The neomycin resistance cassette flanked by FRT was inserted at the XbaI restriction site located between exons 4 and 5. The diphtheria toxin A chain cassette was used as a negative selection marker to ensure homologous recombination. The targeting vector was transfected into the E14Tg2A ES cell line (129/OlaHsd-derived, Baygenomics) and double selected using neomycin and DT-A. To generate chimeras, ES clones were injected into C57BL/6 blastocysts. Chimeric males were bred with C57BL/6 females, and germline transmission of the PLC-β3^neo^ allele was verified by PCR and Southern blotting. PLC-β3^f/f^ mice were generated by crossing PLC-β3^neo^ alleles with a Flp deleter strain (129S4/SvJaSor-*Gt(Rosa)26Sor*^*tm1(FLP1)Dym*^/J), and PLC-β3^∆SMC^ mice were generated by crossing PLC-β3^f/f^ mice with a Cre deleter strain (B6. Cg-Tg(*Tagln-cre*)1Her/J) (Supplementary Fig. 2). NOX1 knockout mice were purchased from The Jackson Laboratory (Bar Harbor, ME, USA). NOX4 knockout mice were generated as previously described^21^. The animals were housed under specific pathogen-free (SPF) conditions, and all animal procedures were performed in accordance with the Animal Care Guidelines issued by the Laboratory Animal Resource Center of Pusan National University School of Medicine after receiving approval from the Pusan National University Institutional Animal Care and Use Committee (mouse: PNU-2019-2259, rat: PNU-2019-2254). The investigation conformed with the Guide for the Care and Use of Laboratory Animals published by the US National Institutes of Health (NIH Publication No. 85-23, revised 1996).

### Carotid artery ligation

Mice were anesthetized with 2.0% isoflurane and placed on a heated surgical pad. To induce neointima, the left common carotid artery (LCCA) was exposed through a small midline incision in the neck. The LCCA was ligated near the carotid bifurcation with a suture, the skin was closed, and the animals were allowed to recover from anesthesia and showed no symptoms of stroke. Four weeks later, the LCCA was isolated. Isolated carotid arteries were fixed in 4% paraformaldehyde at 4 °C overnight and embedded in paraffin. Serial sections (5 µm) were made through the entire carotid artery and stained with hematoxylin & eosin (H&E). The thickness of the neointima samples was visualized by a MIRAX MIDI Versatile Digital Slide Scanner (Carl Zeiss, Jena, Germany). The neointimal area was defined as the area between the luminal circumference and internal elastic lamina. The media area was defined as the area between the internal and external elastic lamina. The neointima/media was defined as the area of the neointima divided by the area of the media. ImageJ software was used for the measurements.

### Measurement of tension

Mouse mesenteric arteries were rapidly removed and immersed in ice-cold physiological salt solution (PSS) (119 mM NaCl, 4.7 mM KCl, 2.5 mM CaCl_2_, 1.17 mM MgSO_4_, 20 mM NaHCO_3_, 1.18 mM KH_2_PO_4_, 0.027 mM EDTA, 11 mM glucose, pH 7.4) in a 95% O_2_ + 5% CO_2_ atmosphere for 1 h at 37 °C_._ The arteries were dissected, and after fat and connective tissues were removed, the tissues were sliced into 2 mm long segments, which were subsequently suspended using two L-shaped stainless-steel wires inserted into the lumen and immersed in PSS buffer in 10 ml organ chambers in a continuously aerated 95% O_2_ + 5% CO_2_ atmosphere. Basal tension was maintained at 0.4 g, and changes in isometric tension were recorded using a force displacement transducer (Grass FT 0.3, Quincy, MA) connected to a power Lab system 400 (ML 118, PowerLab, AD Instruments, Medford, MA). Arteries were allowed to equilibrate for 1 h before measurement, and the chamber solution was changed every 15 min. Inhibitors were added for 20 min prior to AngII stimulation.

### Blood pressure

Male mice (20–25 g, 7 weeks of age) were subcutaneously implanted with an osmotic minipump (Alzet Model 1002, Alza, Vacaville, CA, USA) filled with AngII or NaCl (vehicle-treated mice) for 4 days. The average AngII infusion rate was 2 µg kg^−1^ min^−1^. Blood pressure was measured by tail cuff plethysmography linked with a computerized system (BP2000 Blood Pressure Analysis System, Visitech Systems, Apex, NC, USA).

### Statistical analysis

The data were analyzed and plotted using GraphPad Prism. The unpaired Student’s *t* test (two-tailed) was used to determine the significance of intergroup differences. Multiple sets of data were analyzed by analysis of variance (one-way or two-way ANOVA) with Tukey’s multiple comparison test. Collagen gel contraction assay data were analyzed by nonlinear regression and one-way ANOVA. The results are expressed as the means ± SEMs, and *p* values less than 0.05 were considered significant.

## Results

### Inhibition of PLC or the IP_3_ receptor blocked ROS generation

**Fig. 1:**
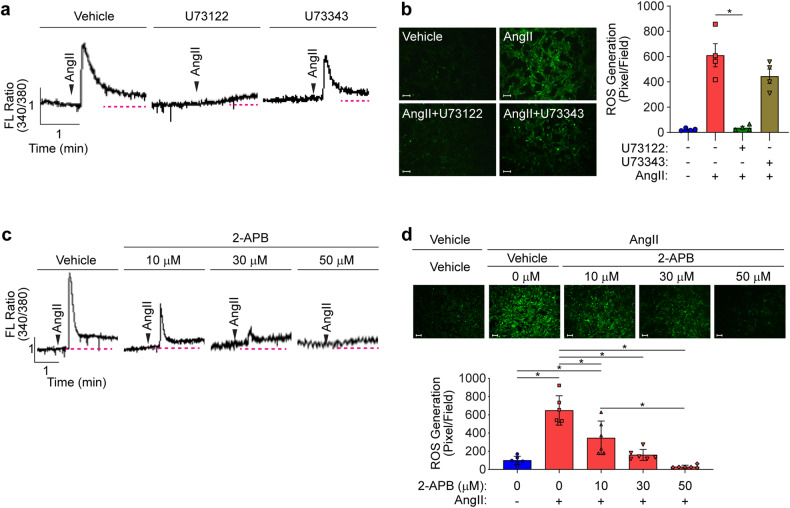
PLC-dependent ROS generation. **a**, **b** VSMCs were pretreated with a pharmacological PLC inhibitor (U73122, 10 μM) and its inactive analog (U73343, 10 μM). AngII-induced calcium mobilization and ROS generation were measured. Arrowheads indicate the time of AngII stimulation. The dotted red line indicates the basal calcium concentration (*n* = 4). **c**, **d** VSMCs were pretreated with the indicated doses of 2-APB for 20 min. AngII-induced calcium mobilization and ROS generation were measured. The dotted red line indicates the basal calcium concentration (*n* = 6). Images were taken using a fluorescence microscope, and ROS levels were quantified by measuring pixel intensities using MetaMorph software. Bar, 100 µm. *p < 0.05. The analysis was conducted using one-way ANOVA followed by Tukey’s multiple comparison test. The data are presented as the mean ± SEM.

As shown in Supplementary Fig. 1a, b, AngII significantly induced ROS, H_2_O_2_, and O_2_^−^ production in VSMCs. We next examined the effect of PLC, which is one of the signaling pathways associated with the AngII receptor. AngII significantly induced intracellular calcium mobilization (Fig. 1a). After an initial rapid increase in calcium mobilization, the intracellular calcium concentration rapidly decreased to a level higher than the basal concentration. Pharmacological inhibition of PLC by U73122 (10 μM) blocked the initial increase, although the intracellular calcium concentration gradually increased. On the other hand, the inactive analog of U73122 (U73343, 10 μM) had no effect. ROS generation was blocked by U73122 but not by its inactive analog (Fig. 1b). To confirm the involvement of calcium mobilization in ROS generation, we next examined the effect of an IP_3_ receptor antagonist (2-APB). Inhibition of the IP_3_ receptor significantly and dose-dependently blocked the initial increase in calcium mobilization, although the intracellular calcium concentration gradually increased (Fig. 1c). Inhibition of the IP_3_ receptor significantly blocked AngII-induced ROS generation (Fig. 1d).

### AngII-induced calcium mobilization and ROS generation are regulated by the PLC-β3 isoform

**Fig. 2:**
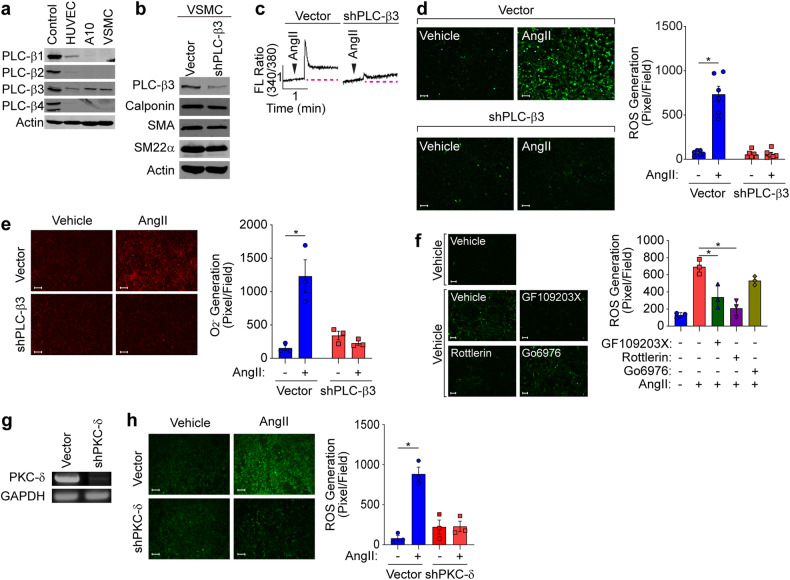
Involvement of PLC-β3 and the PKC-δ isoform in ROS generation. **a** Cell lysates were subjected to western blotting with the indicated antibodies. Rat brain extract was used as a positive control. **b** PLC-β3 was silenced in VSMCs, and the expression of PLC-β3 and contractile marker proteins was confirmed by western blotting. **c** AngII-induced calcium mobilization was determined after PLC-β3 knockdown. The dotted red line indicates the basal calcium concentration. **d** ROS levels were assessed after silencing PLC-β3 (*n* = 6). **e** O_2_^−^ levels were assessed after silencing PLC-β3 (*n* = 3). *p < 0.05. Two-way ANOVA followed by Tukey’s multiple comparison test was used. The results are presented as the means ± SEMs. **f** VSMCs were pretreated with a pan-PKC inhibitor (10 μM), a PKC-δ-specific inhibitor (5 μM) or a PKC-α-specific inhibitor (10 μM) for 20 min, after which AngII-induced ROS levels were measured (*n* = 3). Bar, 100 µm. *p < 0.05. The analysis was conducted using one-way ANOVA followed by Tukey’s multiple comparison test. The results are presented as the means ± SEMs. **g** PKC-δ was silenced in VSMCs, and the expression of PKC-δ was verified. **h** ROS levels were assessed after silencing PLC-δ (*n* = 3). Bar, 100 µm. *p < 0.05. The analysis was conducted using one-way ANOVA followed by Tukey’s multiple comparison test. The data are presented as the mean ± SEM.

Since PLC-β family members are known to be activated by AngII, we next examined the expression of PLC-β isoforms in VSMCs. The major isoform expressed in VSMCs was PLC-β3 (Fig. 2a). PLC-β1 was exclusively expressed in ECs, whereas PLC-β2 and PLC-β4 were not expressed in ECs or VSMCs. Silencing PLC-β3 in VSMCs did not alter the expression of contractile marker genes (Fig. 2b) but significantly attenuated AngII-induced calcium mobilization (Fig. 2c) and ROS generation (Fig. 2d). In addition, AngII significantly induced the generation of O_2_^-^, which was suppressed by silencing PLC-β3 (Fig. 2e). Moreover, AngII-induced ROS generation was significantly attenuated by pretreatment with a pan-PKC inhibitor (GF109203X, 10 µM) or a PKC-δ-specific inhibitor (Rottlerin, 5 µM), whereas inhibition of PKC-α (GO6976, 10 µM) had no effect (Fig. 2f). Furthermore, the silencing of PKC-δ significantly abolished AngII-induced ROS generation (Fig. 2g, h). Finally, ex vivo experiments using aortic tissue fragments showed that disruption of PLC-β3 in VSMCs completely blocked AngII-induced ROS generation (Supplementary Fig. 3a).

### NOX1 mediates AngII-induced ROS generation

**Fig. 3:**
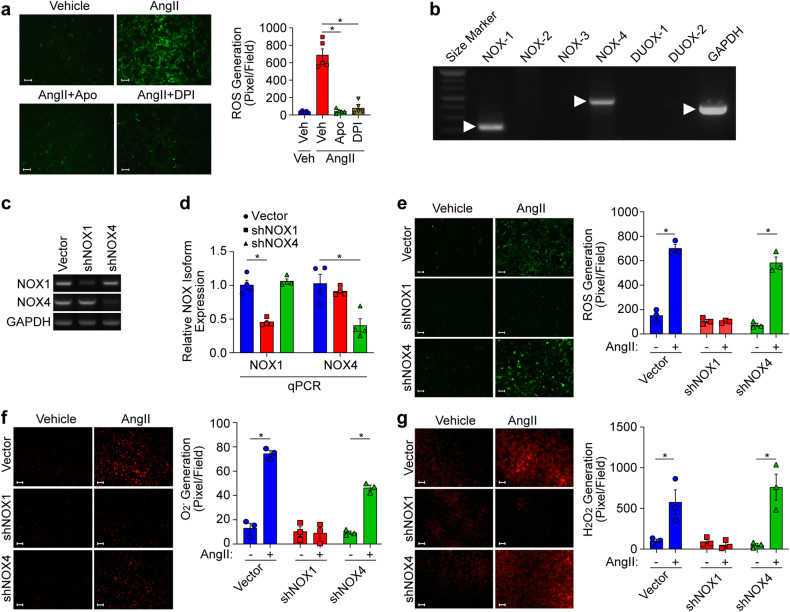
NOX1-dependent ROS generation. **a** AngII-induced ROS generation was measured in VSMCs pretreated with NOX inhibitors (10 μM) for 20 min (*n* = 5). Bar, 100 µm. *p < 0.05. The analysis was performed using one-way ANOVA followed by Tukey’s multiple comparison test. The results are presented as the means ± SEMs. **b** The expression of NOX isoforms in VSMCs was verified by RT‒PCR. **c**, **d** NOX1 or NOX4 was silenced in VSMCs, as confirmed by RT‒PCR and real-time Q‒PCR. (*n* = 4). **e**–**g** ROS, O_2_^−^, and H_2_O_2_ levels were determined after NOX1 or NOX4 silencing. Images were taken using a fluorescence microscope, and ROS, O_2_^−^, and H_2_O_2_ levels were quantified by measuring pixel intensities using MetaMorph software (*n* = 3). Bar, 100 µm. *p < 0.05. The analysis was conducted using two-way ANOVA followed by Tukey’s multiple comparison test. The data are presented as the mean ± SEM.

As NOX reportedly plays a significant role in ROS generation in response to extracellular stimuli, we examined the effects of pharmacologically inhibiting NOX. Pharmacological inhibition of NOX by apocyanin (Apo, 10 μM) or diphenylene iodonium (DPI, 10 μM) completely blocked AngII-induced ROS generation (Fig. 3a). In VSMCs, the NOX1 and NOX4 isoforms were predominantly expressed along with their accessary proteins, such as p22^phox^ and p67^phox^ (Fig. 3b and Supplementary Fig. 4a), and silencing NOX1 significantly reduced AngII-induced ROS generation, whereas silencing NOX4 had no effect (Fig. 3c–e). In addition, the silencing of NOX1 but not NOX4 significantly blocked AngII-induced O_2_^−^ and H_2_O_2_ generation (Fig. 3f, g).

### PLC-β3 and NOX1 are necessary for the AngII-induced proliferation and migration of VSMCs

**Fig. 4:**
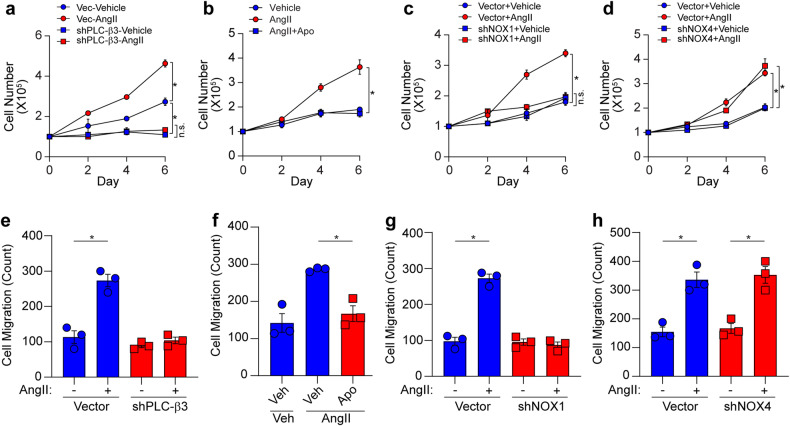
PLC-β3- and NOX1-dependent ROS generation. **a**, **e** AngII-dependent proliferation and migration were determined after silencing PLC-β3 in VSMCs. **b**, **f** VSMCs were pretreated with a NOX inhibitor (Apo, 10 μM) for 20 min and AngII-dependent proliferation an migration were determined. **c**, **d**, **g**, **h** NOX1 or NOX4 was silenced in VSMCs, and the effects on proliferation and migration were assessed (*n* = 3). *p < 0.05. The analysis was conducted using one-way ANOVA followed by Tukey’s multiple comparison test. The data are presented as the mean ± SEM.

Since AngII regulates VSMC proliferation and migration, we next examined the effect of PLC-β3-mediated ROS generation on the proliferation and migration of VSMCs. Silencing PLC-β3, pharmacologically inhibiting NOX, or silencing NOX1, but not NOX4, significantly abrogated the AngII-induced proliferation of VSMCs (Fig. 4a–d). Similarly, AngII-induced VSMC migration was significantly blocked by silencing PLC-β3, pharmacologically inhibiting NOX, or silencing NOX1 (Fig. 4e–h).

### AngII-induced VSMC contraction is not regulated by PLC-β3, NOX1, or NOX4

**Fig. 5:**
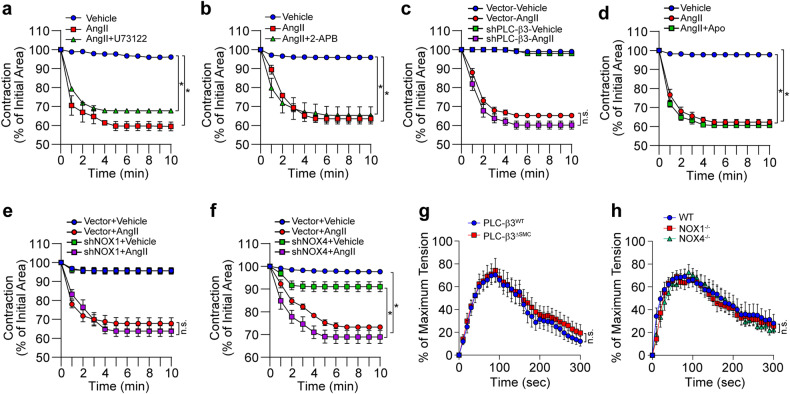
PLC-β3- and NOX-independent vascular contraction. **a**–**c** AngII-dependent vascular contraction was measured after pretreatment of VSMCs with a PLC inhibitor (U73122, 10 μM), an IP_3_ receptor antagonist (2-APB, 50 μM), or PLC-β3-silenced (*n* = 4). **d**–**f** AngII-dependent vascular contraction was measured after pretreating VSMCs with a NOX inhibitor (Apo, 10 μM) or after silencing NOX1 or NOX4 (*n* = 4). **g**, **h** Mesenteric arteries were isolated from PLC-β3^∆SMC^, NOX1^−/−^, or NOX4^−/−^ mice, and AngII-dependent vasoconstriction was measured (*n* = 6). *p < 0.05. The analysis was conducted using one-way ANOVA followed by Tukey’s multiple comparison test. The data are presented as the mean ± SEM.

Pharmacological inhibition of PLC (U73122, 10 μM) or the IP_3_ receptor (2-APB, 50 μM) did not affect AngII-induced VSMC contraction (Fig. 5a, b), and silencing PLC-β3 (Fig. 5c), inhibiting NOX activity (Fig. 5d), and silencing NOX1 (Fig. 5e) or NOX4 (Fig. 5f) had little effect on AngII-induced VSMC contraction. To further confirm the role of PLC-β3 and NOX1/4 in arterial constriction, we established VSMC-specific knockout (KO) of PLC-β3 (Supplementary Fig. 2a–d) and whole-body knockout (KO) of NOX1 and NOX4 in animal models (Supplementary Fig. 2e). Compared with those of wild-type mice, AngII-induced vasoconstriction of mesenteric arteries isolated from PLC-β3^∆SMC^ (Fig. 5g), NOX1^−/−^, and NOX4^−/−^ mice (Fig. 5h) showed subtle differences. In addition, deletion of the PLC-β3 and NOX1 genes did not alter VSMC marker gene expression (Supplementary Fig. 2c, f). Furthermore, ablation of PLC-β3 in VSMCs did not affect AngII-induced vasoconstriction of the aortic arteries (Supplementary Fig. 3b).

### ROCK2, MLCK, and L-type calcium channels are involved in AngII-induced VSMC contraction

**Fig. 6:**
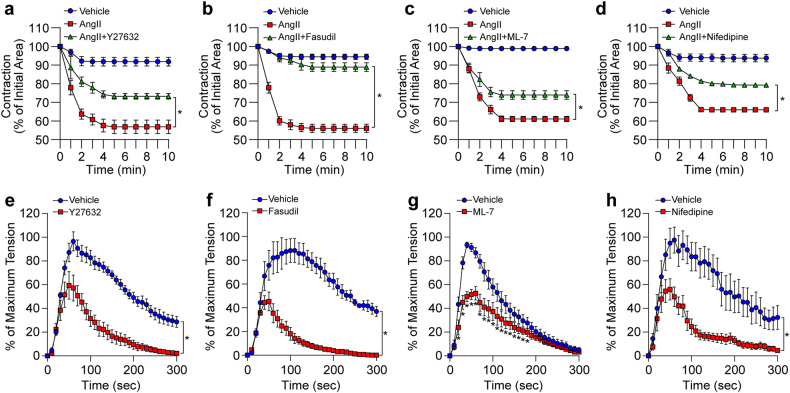
ROCK-, MLCK-, and L-type calcium channel-dependent vasoconstriction. **a**–**d** AngII-dependent vascular contraction was measured after pretreatment of VSMCs with a pan ROCK inhibitor (Y27632, 5 μM), a selective ROCK2 inhibitor (fasudil, 50 μM), an MLCK inhibitor (ML-7, 10 μM), or an L-type calcium channel inhibitor (nifedipine, 20 μM) for 20 min (*n* = 4). **e**–**h** Mesenteric arteries were isolated from mice, and AngII-induced vasoconstriction was measured in the presence or absence of a pan ROCK inhibitor (5 μM), a selective ROCK2 inhibitor (50 μM), an MLCK inhibitor (10 μM), or an L-type calcium channel inhibitor (nifedipine, 20 μM) (*n* = 6). *p < 0.05. The analysis was conducted using one-way ANOVA followed by Tukey’s multiple comparison test. The data are presented as the mean ± SEM.

Since PLC-β3/NOX1 signaling cascades were not involved in AngII-induced VSMC contraction (Fig. 5), we examined the effects of other signaling cascades. Inhibition of ROCK by Y27632 (10 μM) or fasudil (50 μM) significantly blocked AngII-induced VSMC contraction (Fig. 6a, b). In addition, inhibition of MLCK by ML-7 (10 μM) (Fig. 6c) or inhibition of L-type calcium channels by nifedipine (20 μM) (Fig. 6d) or nimodipine (10 μM) (Supplementary Fig. 3c) significantly attenuated AngII-induced VSMC contraction. Similarly, the inhibition of ROCK (Fig. 6e, f), MLCK (Fig. 6g), or L-type calcium channels (Fig. 6h) significantly attenuated the AngII-induced vasoconstriction of mesenteric arteries, and the AngII-induced vasoconstriction of aortic arteries was significantly attenuated by inhibiting ROCK (Supplementary Fig. 3d).

### ROCK2 and MLCK activation requires extracellular calcium but PLC-β3 does not

**Fig. 7:**
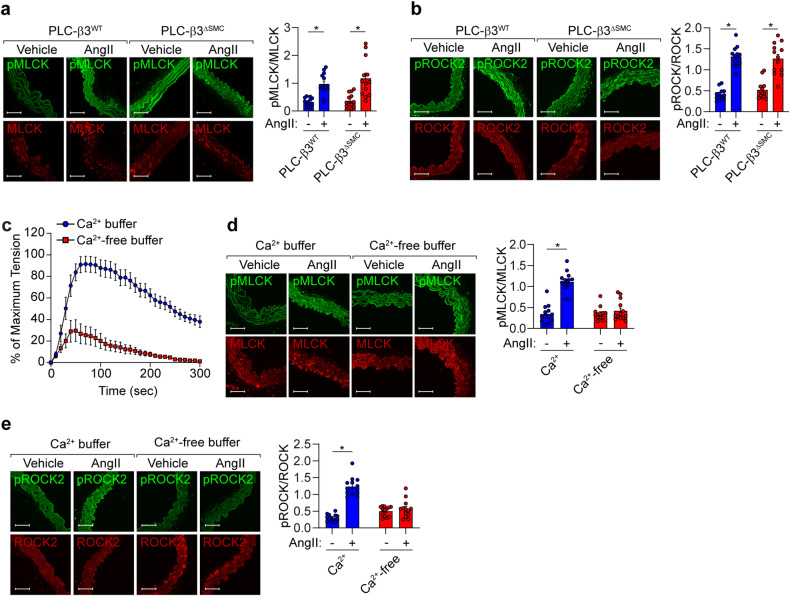
Extracellular calcium-dependent phosphorylation of MLCK and ROCK2. **a**, **b** Aortic tissues were isolated from PLC-β3^∆SMC^ mice, and the AngII-dependent phosphorylation of MLCK and ROCK2 was measured (*n* = 13). **c** AngII-dependent vasoconstriction was measured in the presence (Ca^2+^ buffer) or absence (Ca^2+^-free buffer) of extracellular calcium (*n* = 6). **d**, **e** AngII-dependent phosphorylation of MLCK and ROCK2 was measured in the presence or absence of extracellular calcium (*n* = 13). Images were taken using a fluorescence microscope, and phosphorylation was quantified by measuring pixel intensities using MetaMorph software. Bar, 50 µm. *p < 0.05. The analysis was conducted using two-way ANOVA followed by Tukey’s multiple comparison test. The data are presented as the mean ± SEM.

Since calcium mobilization is necessary for the activation of MLCK and ROCK2, we next examined the calcium sources that activate MLCK and ROCK2. Ablation of PLC-β3 in VSMCs did not affect the AngII-induced activation of MLCK or ROCK2 (Fig. 7a, b). AngII-induced vasoconstriction of mesenteric arteries and the activation of MLCK and ROCK2 were significantly blocked in the absence of extracellular calcium (Fig. 7c–e).

### PLC-β3 and NOX1 are not required for AngII-dependent blood pressure regulation but are required for neointima formation

**Fig. 8:**
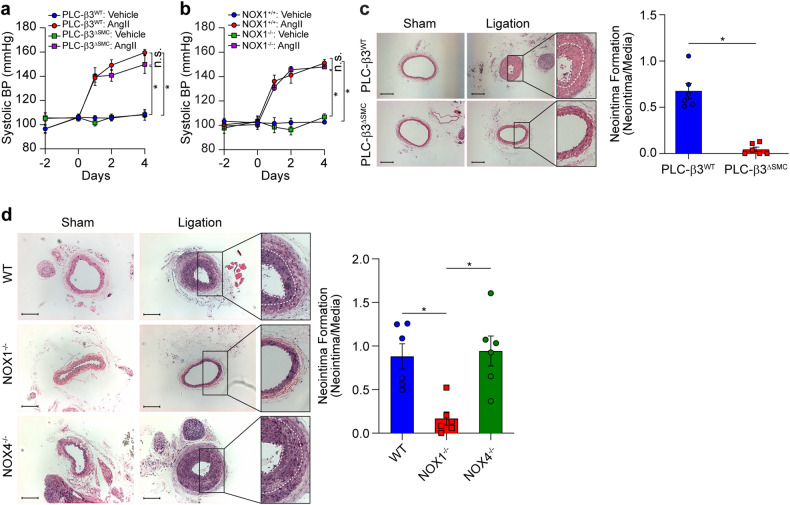
PLC-β3- and NOX1-dependent neointima formation. **a**, **b** Systolic blood pressure was measured in PLC-β3^∆SMC^ and NOX1^−/−^ mice after the administration of AngII (2 µg kg^−1^ min^−1^) (*n* = 6). *p < 0.05. The analysis was conducted using one-way ANOVA followed by Tukey’s multiple comparison test. The results are presented as the means ± SEMs. **c**, **d** The left common carotid arteries of PLC-β3^∆SMC^, NOX1^−/−^ and NOX4^−/−^ mice were ligated for 4 weeks and stained with hematoxylin and eosin. The right common carotid arteries were used as sham-operated controls. White dashed line, neointimal area (*n* = 6). The results are presented as neointimal to medial area ratios. Bar, 100 µm. *p < 0.05. **c**, **d** The analysis was conducted using an unpaired *t-*test (two-tailed) and one-way ANOVA followed by Tukey’s multiple comparison test. The data are presented as the mean ± SEM.

Since PLC-β3/NOX1-mediated ROS generation was found to be necessary for the proliferation and migration of VSMCs but not for VSMC contraction, we examined the effects of PLC-β3 and NOX1 on the regulation of blood pressure and neointima formation. Ablation of PLC-β3 in VSMCs did not affect blood pressure regulation by the infusion of AngII (2 µg/kg/min) (Fig. 8a). Furthermore, blood pressure regulation by AngII was unaffected in mice lacking NOX1 (Fig. 8b). Ligation-induced neointima formation was completely blocked in PLC-β3^∆SMC^ mice (Fig. 8c) and in mice lacking NOX1 but not in mice lacking NOX4 (Fig. 8d).

## Discussion

The present study delineates the roles of PLC-β in the AngII-induced physiological responses of VSMCs, such as vascular proliferation, migration, and contraction. PLC-β3 enhances calcium mobilization upon AngII stimulation and results in the production of ROS through NOX1, thus enhancing VSMC proliferation and migration. On the other hand, AngII-induced vascular contraction is regulated by ROCK and MLCK, which are activated by extracellular calcium mobilization rather than intracellular calcium mobilization by PLC-β3. Indeed, mice lacking PLC-β3 or NOX1 exhibited normal blood pressure regulation by AngII, whereas ligation-induced neointima formation, which mimics vascular proliferation and migration, was significantly blocked. Although our data suggests that NOX1 is not involved in blood pressure regulation, a recent report suggested that both NOX1 and NOX4 are involved in AngII-induced ROS generation and contraction in VSMCs^22^. However, we would like to emphasize that several different experimental conditions, such as the observational time window of blood pressure regulation and the genetic background of the animal model, could lead to different experimental results.

Our results emphasize the crucial role of ROS in the proliferation and migration of VSMCs. ROS production imbalances are closely linked to many pathological responses, such as the regulation of vasotone and immune responses^23^. In particular, AngII, which primarily acts on VSMCs, regulates the physiology of various VSMCs and stimulates ROS production by activating NOX^11^. Notably, VSMCs expressed only the PLC-β3 isoform and the NOX1 and NOX4 isoforms of NOX (Fig. 2a and Supplementary Fig. 2). Our observation suggests that AngII-induced ROS production is mediated by the PLC-β3/NOX1 signaling axis since silencing PLC-β3 or NOX1 but not NOX4 impeded AngII-induced ROS production (Figs. 2d and 3e). Although the mechanism underlying the AngII-induced activation of NOX1 remains ambiguous, a previous reconstitution experiment revealed that NOXA1, NOXO1, and PLC-β-activated PKC activity are required for NOX1 activation and the subsequent production of ROS^24^. Consistent with these findings, our results showed that selective inhibition of PKC-δ suppressed AngII-induced ROS production (Fig. 2e). Further study is required to elucidate the nature of the interplay between PKC-δ and NOX1 during the production of ROS.

Increasing evidence indicates that ROS are involved in the proliferation and migration of VSMCs. AngII is a growth factor that can activate tyrosine kinases and mitogen-activated protein kinases (MAPKs) and induce the expression of proto-oncogenes^7^, which suggests that ROS modulate signal transduction cascades involved in proliferation and migration. Indeed, ROS have been shown to mediate VSMC proliferation via the PKC-dependent activation of ERK1/2^25,26^ and to stimulate VSMC proliferation via the expression of oncogenes such as *c-myc*, *c-fos*, and *c-jun*^27,28^. It has also been suggested that the ROS-induced proliferation of VSMCs is mediated by the dominant negative helix-loop-helix protein Id3 and cyclophilin A (CyPA)^29–31^. Currently, the mechanism by which ROS regulate signaling molecules remains unclear. However, amino acid residues in proteins, especially cysteine, are readily oxidized by superoxide and hydrogen peroxide^32^, which alters protein functions. Moreover, it was recently demonstrated that platelet-derived growth factor receptor (PDGFR)-induced hydrogen peroxide generation oxidizes cysteine residues of phosphatases and thereby maintains tyrosine kinase signaling cascades^33^. Similarly, AngII-induced ROS generation might modify and stimulate signaling molecules involved in the proliferation and migration of VSMCs.

We provide evidence that PLC-β3 is not involved in VSMC contraction in vitro, mesenteric artery constriction ex vivo, or blood pressure regulation in vivo. AngII stimulation upregulates intracellular calcium in a biphasic manner, characterized by an initial rapid but transient response followed by a slow, tonic response^34^. It has been suggested that the rapid transient response is mediated by calcium mobilization from intracellular calcium stores regulated by PLC and that the subsequent tonic response is mediated by calcium influx mainly regulated by voltage-gated calcium channels (VGCCs)^35^. However, our data showed that AngII-induced VSMC contraction was not affected by pharmacological inhibition or gene silencing of PLC-β3 (Fig. 5a–c) but that the rapid transient response was significantly abolished (Figs. 1a and 2c). In addition, the vascular tone of mesenteric arteries from PLC-β3^∆SMC^ mice was altered after AngII stimulation (Fig. 5g). Currently, the mechanism responsible for the regulation of the tonic intracellular calcium phase by AngII in the absence of PLC-β3 is ambiguous. One explanation offered to reconcile these increases during the tonic phase in the absence of PLC-β3 is that the AT_1_ receptor directly triggers chloride anion channels and thereby changes the resting membrane potential and induces the activation of VGCCs^36^. Therefore, it is possible that AngII induces the slow, tonic phase of intracellular calcium and thus vascular contraction in the absence of PLC-β3.

MLCK is a key player in the contraction of VSMCs, and its activation was not affected by PLC-β3 (Fig. 7a). On the other hand, the depletion of extracellular calcium significantly blocked its activation (Fig. 7d). These data suggest that calcium influx rather than calcium mobilization by PLC-β3 plays an indispensable role in vascular contraction and MLCK activation. MLC phosphorylation levels are also regulated by proteins that sense transient changes in calcium ion concentrations (calcium sensitization)^37^. The AT_1_ receptor downregulates MLCP activity by phosphorylating it at Thr^695^ via the activation of ROCK, which results in the sensitization of MLC phosphorylation to intracellular calcium^38^. ROCK is the upstream regulator of MLCP and is activated by calcium influx and G_12/13_^39,40^. Consistent with these previous reports, our results also showed that in VSMCs, ROCK activation was abolished in the absence of extracellular calcium, whereas the null mutation of PLC-β3 did not negate ROCK activation (Fig. 7b, e). Notably, G_12/13_, rather than G_q_, which is coupled to PLC activation, is involved in the regulation of VSMC contraction^41^. Therefore, we hypothesize that the AT_1_ receptor regulates vascular contraction by signaling the VGCC-induced activation of MLCK during the tonic phase after AngII stimulation and that this effect is enhanced by calcium sensitization mediated by G_12/13_ and the VGCC and subsequent ROCK activation.

Currently, there are thought to be many different contraction mechanisms of smooth muscle cells in response to various ligands, unlike those of skeletal muscle cells. More importantly, the precise underlying mechanism of each ligand-induced contraction of smooth muscle cells is still unclear. For example, the pattern of myographic contraction between AngII and norepinephrine is quite different, although both ligands share PLC-β and calcium elevation, which are common signaling cascades^42^. In particular, AngII-induced contraction exhibited a single bell-shaped pattern; however, norepinephrine-induced contraction exhibited a sustained pattern. It is also notable that AngII-induced calcium mobilization is relatively weak (∆[Ca^2+^]i, 60–70 nM) and shows a tonic phase of calcium elevation, whereas norepinephrine-induced calcium mobilization is more robust (∆[Ca^2+^]i, 300 nM) than that of AngII^36,43^. It is still unclear whether these weak elevations in intracellular calcium and the tonic phase of calcium mobilization induced by AngII are responsible for VSMC contraction in the absence of PLC-β3. However, it has been reported that the AT1 receptor is coupled to VGCCs, thereby regulating the tonic phase of calcium elevation^36^. Similarly, we also provide evidence that disruption of intracellular calcium mobilization by null mutation of PLC-β3 is dispensable for vessel contraction (Fig. 7a) and that extracellular calcium is required for MLCK phosphorylation (Fig. 7d), which is a key response during VSMC contraction. Since AngII-induced calcium mobilization is relatively weak compared with that induced by norepinephrine, additional signaling cascades, which are also called calcium sensitization, may be required for maintaining MLCK phosphorylation. Recent reports suggest that ROCK, which is activated by G12/13, stabilizes MLCK phosphorylation by inactivating MLCP, thereby conferring calcium sensitization^38^. Therefore, we suggest that PLC-β3-mediated transient and weak levels of intracellular calcium mobilization are not sufficient to induce MLCK phosphorylation; however, the tonic phase of calcium elevation and calcium sensitization signaling, which are mediated by G12/13-mediated ROCK activation, are needed. In line with these suggestions, we provide clear evidence that PLC-β3 is dispensable for VSMC contraction and blood vessel constriction, that extracellular calcium is required for blood vessel constriction and MLCK phosphorylation, and that ROCK is required for VSMC contraction and blood vessel constriction. Importantly, this study provides direct evidence regarding PLC-β3 and blood vessel constriction by using a tissue-specific knockout animal model.

It is very difficult to define the major extracellular stimuli that cause pathological consequences in many different surgical models, such as carotid artery ligation and balloon injury mouse models. However, we emphasize that AngII-induced arterial constriction and blood pressure regulation were not mediated by PLC-β3 (Fig. 8a, b), whereas AngII-induced ROS generation and proliferation were exclusively dependent on PLC-β3 (Figs. 2–4). Notably, we did not observe basal proliferation in VSMCs in which PLC-β3 was silenced (Fig. 4a), which indicates that PLC-β3 is an essential mediator of the intrinsic proliferative capacity of VSMCs. Therefore, we suggest that the attenuation of neointima formation in mice lacking PLC-β3 could result from the loss of the intrinsic proliferation capacity of VSMCs, although AngII may not be the major leading cause of VSMC proliferation during carotid artery ligation-induced neointima formation.

Our data indicate that PLC-β3 is required for AngII-induced VSMC proliferation and migration but not for VSMC contraction. PLC-β3-dependent intracellular calcium mobilization was found to be necessary for NOX activation and subsequent ROS production, and PLC-β3-dependent ROS production was found to be required for VSMC proliferation and migration. Furthermore, VGCC-dependent calcium influx was required for the activation of MLCK or ROCK, which led to VSMC contraction, whereas PLC-β3 was not required for the activation of MLCK, ROCK, or VSMC contraction.

### Supplementary information

Supplementary File

## References

[1] Eguchi, S., Kawai, T., Scalia, R. & Rizzo, V. Understanding angiotensin II type 1 receptor signaling in vascular pathophysiology. *Hypertension* **71**, 804–810 (2018). 29581215 10.1161/HYPERTENSIONAHA.118.10266 PMC5897153

[2] Singh, K. D. & Karnik, S. S. Angiotensin receptors: structure, function, signaling and clinical applications. *J. Cell Signal.* **1**, 111 (2016). 27512731 10.4172/jcs.1000111 PMC4976824

[3] Rattan, S. Ca2+/calmodulin/MLCK pathway initiates, and RhoA/ROCK maintains, the internal anal sphincter smooth muscle tone. *Am. J. Physiol. Gastrointest. Liver Physiol.* **312**, G63–G66 (2017). 27932502 10.1152/ajpgi.00370.2016 PMC5283903

[4] Pereira da Silva, E. A., Martin-Aragon Baudel, M., Navedo, M. F. & Nieves-Cintron, M. Ion channel molecular complexes in vascular smooth muscle. *Front. Physiol.* **13**, 999369 (2022). 36091375 10.3389/fphys.2022.999369 PMC9459047

[5] Touyz, R. M. et al. Vascular smooth muscle contraction in hypertension. *Cardiovasc. Res.* **114**, 529–539 (2018). 29394331 10.1093/cvr/cvy023 PMC5852517

[6] Muranyi, A. et al. Phosphorylation of Thr695 and Thr850 on the myosin phosphatase target subunit: inhibitory effects and occurrence in A7r5 cells. *FEBS Lett.* **579**, 6611–6615 (2005). 16297917 10.1016/j.febslet.2005.10.055

[7] Forrester, S. J. et al. Angiotensin II signal transduction: an update on mechanisms of physiology and pathophysiology. *Physiol. Rev.* **98**, 1627–1738 (2018). 29873596 10.1152/physrev.00038.2017 PMC6335102

[8] Cocco, L., Follo, M. Y., Manzoli, L. & Suh, P. G. Phosphoinositide-specific phospholipase C in health and disease. *J. Lipid Res.* **56**, 1853–1860 (2015). 25821234 10.1194/jlr.R057984 PMC4583093

[9] Mehta, P. K. & Griendling, K. K. Angiotensin II cell signaling: physiological and pathological effects in the cardiovascular system. *Am. J. Physiol. Cell Physiol.* **292**, C82–C97 (2007). 16870827 10.1152/ajpcell.00287.2006

[10] Mohamed, R. et al. GPCR transactivation signalling in vascular smooth muscle cells: role of NADPH oxidases and reactive oxygen species. *Vasc. Biol.* **1**, R1–R11 (2019). 32923966 10.1530/VB-18-0004 PMC7439842

[11] Lassegue, B., San Martin, A. & Griendling, K. K. Biochemistry, physiology, and pathophysiology of NADPH oxidases in the cardiovascular system. *Circ. Res.* **110**, 1364–1390 (2012). 22581922 10.1161/CIRCRESAHA.111.243972 PMC3365576

[12] Cave, A. C. et al. NADPH oxidases in cardiovascular health and disease. *Antioxid. Redox Signal.* **8**, 691–728 (2006). 16771662 10.1089/ars.2006.8.691

[13] Lassegue, B. & Griendling, K. K. NADPH oxidases: functions and pathologies in the vasculature. *Arterioscler. Thromb. Vasc. Biol.* **30**, 653–661 (2010). 19910640 10.1161/ATVBAHA.108.181610 PMC2841695

[14] Krause, K. H. Tissue distribution and putative physiological function of NOX family NADPH oxidases. *Jpn. J. Infect. Dis.* **57**, S28–S29 (2004). 15507765

[15] Lyle, A. N. et al. Poldip2, a novel regulator of Nox4 and cytoskeletal integrity in vascular smooth muscle cells. *Circ. Res.* **105**, 249–259 (2009). 19574552 10.1161/CIRCRESAHA.109.193722 PMC2744198

[16] Bedard, K. & Krause, K. H. The NOX family of ROS-generating NADPH oxidases: physiology and pathophysiology. *Physiol. Rev.* **87**, 245–313 (2007). 17237347 10.1152/physrev.00044.2005

[17] Gupte, S. A. et al. Peroxide generation by p47phox-Src activation of Nox2 has a key role in protein kinase C-induced arterial smooth muscle contraction. *Am. J. Physiol. Heart Circ. Physiol.* **296**, H1048–H1057 (2009). 19168729 10.1152/ajpheart.00491.2008 PMC2670684

[18] Oro, C., Qian, H. & Thomas, W. G. Type 1 angiotensin receptor pharmacology: signaling beyond G proteins. *Pharmacol. Ther.* **113**, 210–226 (2007). 17125841 10.1016/j.pharmthera.2006.10.001 PMC7112676

[19] Zhnag, M., Goforth, P. & Satin, L. The Ca^2+^ dynamics of isolated mouse *β*-cells and islets: implicatios for mathematical models. *Biophys. J.* **84**, 2852–2870 (2003). 12719219 10.1016/S0006-3495(03)70014-9 PMC1302850

[20] Jeon, Y. et al. TopBP1 deficiency causes an early embryonic lethality and induces cellular senescence in primary cells. *J. Biol. Chem.* **286**, 5414–5422 (2011). 21149450 10.1074/jbc.M110.189704 PMC3037654

[21] Lee, J. H. et al. Interaction of NADPH oxidase 1 with Toll-like receptor 2 induces migration of smooth muscle cells. *Cardiovasc. Res.* **99**, 483–493 (2013). 23749776 10.1093/cvr/cvt107

[22] Park, J. M. et al. NADPH oxidase 1 mediates acute blood pressure response to angiotensin II by contributing to calcium influx in vascular smooth muscle cells. *Arterioscler. Thromb. Vasc. Biol.* **42**, e117–e130 (2022). 35354309 10.1161/ATVBAHA.121.317239

[23] Chen, Q., Wang, Q., Zhu, J., Xiao, Q. & Zhang, L. Reactive oxygen species: key regulators in vascular health and diseases. *Br. J. Pharmacol.* **175**, 1279–1292 (2018). 28430357 10.1111/bph.13828 PMC5867026

[24] Choi, H. et al. Mechanism of angiotensin II-induced superoxide production in cells reconstituted with angiotensin type 1 receptor and the components of NADPH oxidase. *J. Biol. Chem.* **283**, 255–267 (2008). 17981802 10.1074/jbc.M708000200

[25] Jiang, R. et al. Protein kinase Calpha stimulates hypoxia‑induced pulmonary artery smooth muscle cell proliferation in rats through activating the extracellular signal‑regulated kinase 1/2 pathway. *Mol. Med. Rep.* **16**, 6814–6820 (2017). 28901444 10.3892/mmr.2017.7478 PMC5865839

[26] Schauwienold, D. et al. ERK1/2-dependent contractile protein expression in vascular smooth muscle cells. *Hypertension* **41**, 546–552 (2003). 12623957 10.1161/01.HYP.0000054213.37471.84

[27] Dong, L. et al. Mxi1-0 promotes hypoxic pulmonary hypertension via ERK/c-Myc-dependent proliferation of arterial smooth muscle cells. *Front. Genet.* **13**, 810157 (2022). 35401684 10.3389/fgene.2022.810157 PMC8984142

[28] Kong, L. et al. PKCbeta promotes vascular inflammation and acceleration of atherosclerosis in diabetic ApoE null mice. *Arterioscler. Thromb. Vasc. Biol.* **33**, 1779–1787 (2013). 23766264 10.1161/ATVBAHA.112.301113 PMC3865290

[29] Jin, Z. G. et al. Cyclophilin A is a secreted growth factor induced by oxidative stress. *Circ. Res.* **87**, 789–796 (2000). 11055983 10.1161/01.RES.87.9.789

[30] Nickenig, G. et al. Redox-sensitive vascular smooth muscle cell proliferation is mediated by GKLF and Id3 in vitro and in vivo. *FASEB J.* **16**, 1077–1086 (2002). 12087069 10.1096/fj.01-0570com

[31] Satoh, K., Nigro, P. & Berk, B. C. Oxidative stress and vascular smooth muscle cell growth: a mechanistic linkage by cyclophilin A. *Antioxid. Redox Signal.* **12**, 675–682 (2010). 19747062 10.1089/ars.2009.2875 PMC2861539

[32] MacKay, C. E. & Knock, G. A. Control of vascular smooth muscle function by Src-family kinases and reactive oxygen species in health and disease. *J. Physiol.* **593**, 3815–3828 (2015). 25384773 10.1113/jphysiol.2014.285304 PMC4575571

[33] Choi, M. H. et al. Regulation of PDGF signalling and vascular remodelling by peroxiredoxin II. *Nature* **435**, 347–353 (2005). 15902258 10.1038/nature03587

[34] Rhee, S. G., Woo, H. A. & Kang, D. The role of peroxiredoxins in the transduction of H(2)O(2) signals. *Antioxid. Redox Signal.* **28**, 537–557 (2018). 28587524 10.1089/ars.2017.7167

[35] Wynne, B. M., Chiao, C. W. & Webb, R. C. Vascular smooth muscle cell signaling mechanisms for contraction to angiotensin II and endothelin-1. *J. Am. Soc. Hypertens.* **3**, 84–95 (2009). 20161229 10.1016/j.jash.2008.09.002 PMC2704475

[36] Fuller, A. J. et al. Calcium and chloride channel activation by angiotensin II-AT1 receptors in preglomerular vascular smooth muscle cells. *Am. J. Physiol. Renal Physiol.* **289**, F760–F767 (2005). 15942047 10.1152/ajprenal.00422.2004 PMC1314975

[37] Perrino, B. A. Calcium sensitization mechanisms in gastrointestinal smooth muscles. *J. Neurogastroenterol. Motil.* **22**, 213–225 (2016). 26701920 10.5056/jnm15186 PMC4819859

[38] Sakurada, S. et al. Ca2+-dependent activation of Rho and Rho kinase in membrane depolarization-induced and receptor stimulation-induced vascular smooth muscle contraction. *Circ. Res.* **93**, 548–556 (2003). 12919947 10.1161/01.RES.0000090998.08629.60

[39] Nunes, K. P. & Webb, R. C. New insights into RhoA/Rho-kinase signaling: a key regulator of vascular contraction. *Small GTPases* **12**, 458–469 (2021). 32970516 10.1080/21541248.2020.1822721 PMC8583239

[40] Strassheim, D. et al. RhoGTPase in vascular disease. *Cells* **8**, 551 (2019). 31174369 10.3390/cells8060551 PMC6627336

[41] Gohla, A., Schultz, G. & Offermanns, S. Role for G(12)/G(13) in agonist-induced vascular smooth muscle cell contraction. *Circ. Res.* **87**, 221–227 (2000). 10926873 10.1161/01.RES.87.3.221

[42] Trufanov, S. K. et al. The role of two-pore channels in norepinephrine-induced [Ca(2+)](i) rise in rat aortic smooth muscle cells and aorta contraction. *Cells* **8**, 1144 (2019). 10.3390/cells8101144 PMC6829401 31557916

[43] Dora, K. A., Doyle, M. P. & Duling, B. R. Elevation of intracellular calcium in smooth muscle causes endothelial cell generation of NO in arterioles. *Proc. Natl Acad. Sci. USA* **94**, 6529–6534 (1997). 9177252 10.1073/pnas.94.12.6529 PMC21084

