# Supplementary material for: Phospholipase C-β3 is dispensable for vascular constriction but indispensable for vascular hyperplasia
Source: Exp Mol Med. 2024 Jul 1;56(7):1620–30. doi: 10.1038/s12276-024-01271-6 (PMC11297146; doi:10.1038/s12276-024-01271-6)
Supplement: Supplementary file 1 — Supplementary File [file 12276_2024_1271_MOESM1_ESM.pdf]

# Supplementary Figures

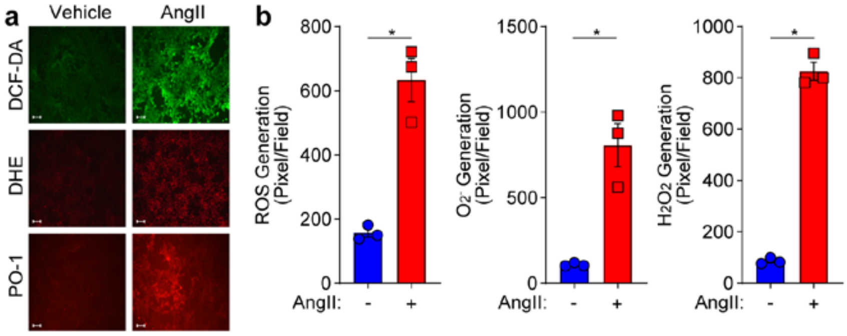


**Online Figure S1 Jin et al.**

**Supplementary Fig. 1 AngII-induced ROS generaion. a, b** AngII-induced ROS, $O_2^-$, and $H_2O_2$ were measured in VSMCs. Images were taken using a fluorescence microscope, ROS, $O_2^-$, and $H_2O_2$ levels were quantified by measuring pixel intensities using MetaMorph software (n = 3). Bar, 100 µm. * $P$<0.05. The analysis was conducted using two-way ANOVA followed by Tukey's multiple comparison test. The data are presented as the mean ± SEM.

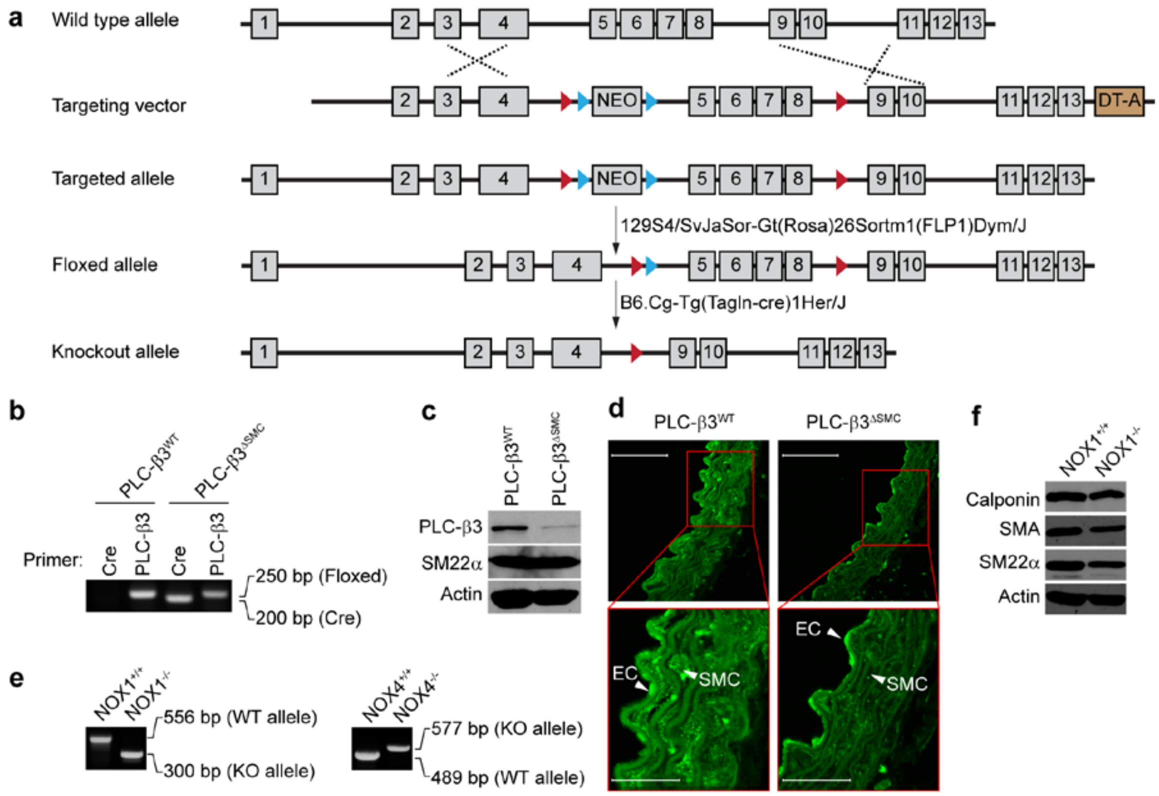


**Online Figure S2 Jin et al.**

**Supplementary Fig. 2 Verification knockout animals. a** Schematic representation of tissue-specific knockout of PLC-β3. **b** Tail tissues of PLC-β3 $^{WT}$ and PLC-β3$^{\Delta SMC}$ mice were lysed and amplified with specific primers (Supplementary Table 1). The 200 bp band indicated the *Cre* allele, the 250 bp band indicated PLC-β3 floxed allele. **c** VSMCs were isolated from PLC-β3 $^{WT}$ and PLC-β3$^{\Delta SMC}$ mice, and expression of PLC-β3, SM22α were verified by western blot analysis. **d** Aortic tissues were isolated from PLC-β3 $^{WT}$ and PLC-β3$^{\Delta SMC}$ mice, and stained with PLC-β3. Arrow heads indicate EC or SMC. Representative images were captured using a confocal microscope. Bar, 50 μm. **e** Tail tissues form both NOX1 and NOX4 knockout mice were genotyped by using specific primers (Supplementary Table 1). PCR products from both wild type (WT) and knockout (KO) alleles were indicated. **f** VSMCs were isolated from

$NOX1^{+/+}$ and $NOX1^{-/-}$ mice, and expression of calponin, SMA, SM22α were verified by western blot analysis.

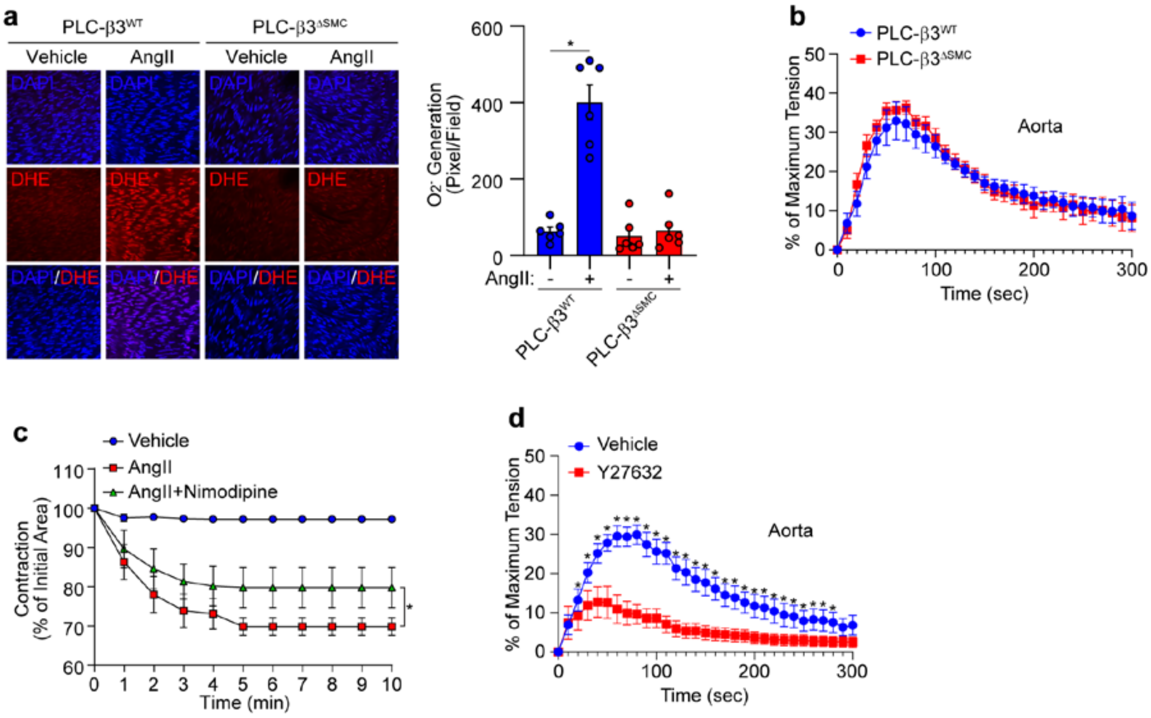


**Online Figure S3 Jin et al.**

**Supplementary Fig. 3 Regulation of aortic tissue constriction by L-Type calcium channel and ROCK but not by PLC-β3. a** Aortas were isolated from either PLC-β3$^{WT}$ or PLC-β3$^{\Delta SMC}$ mice, and AngII-induced ROS were measured in aortas. Images were taken using a fluorescence microscope, ROS levels were quantified by measuring pixel intensities using MetaMorph software (n = 6). **b** Aortic rings were isolated from either PLC-β3$^{WT}$ or PLC-β3$^{\Delta SMC}$ mice, and AngII-dependent vasoconstriction was measured (n = 6). **c** VSMCs were pretreated with the L-type calcium channel (20 μM) for 20 min, and AngII-dependent contraction was measured (n = 4). **d** Aortic rings were isolated form wild type mice, and AngII-induced vasoconstriction was measured in the presence or absence of Rho-kinase inhibitor (10 μM) (n = 6). * *P*<0.05. One-way ANOVA followed by Tukey's multiple comparison test. The

data are presented as the mean ± SEM.

a

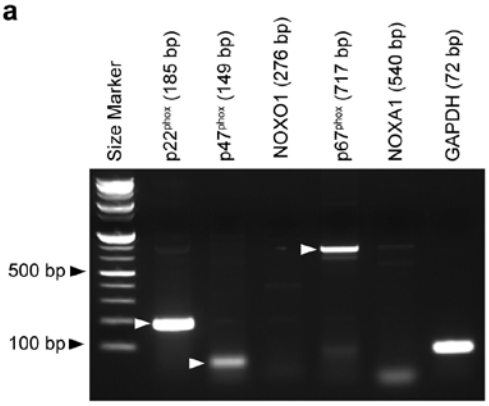


**Online Figure S4 Jin et al.**

**Supplementary Fig. 4 mRNA expression of NOX1 accessory proteins in VSMCs.**

**a** Expression of NOX1 accessory proteins in VSMCs was verifed by RT-PCR. Arrows indicate expected PRC product of each accessary protein.

# Supplementary Tables

## Supplementary Table 1. Primer sequence information for genotyping.

| Gene | Forward (5’->3’) | Reverse (5’->3’) |
|---|---|---|
| Floxed PLC-β3 | ccaatggttgtgtgggacctgc | tcaacattgtcaggcagaagacag |
| Cre | tgccaggatcagggttaaagata | acccggcaaaacaggtagttatt |
| NOX1, wild type | ttgcagttgttgggtgatct | tagctgccatggaactgag |
| NOX1, targeted | cgagcgctctgaagttcct | tagctgccatggaactgag |
| NOX4, wild type | tctctgatgagttaccaagg | gagcaaggacactaacgtag |
| NOX4, targeted | cgaatatcatggtggaaaat | gagcaaggacactaacgtag |

## Supplementary Table 2. Primer sequence information for RT-PCR.

| Gene | Forward (5’->3’) | Reverse (5’->3’) |
|---|---|---|
| NOX1 | cttcctcactggctcggata | cgacagcatttgcgcaggct |
| NOX2 | tgactcggttggctggcatc | cgcaaaggtacaggaacatggg |
| NOX3 | ttgtcacacttgttcaacctgg | tcacacgcatacaagaccacagga |
| NOX4 | cttaaacacctctgtctgcttg | cacctgtcaggcccggaaca |
| NOX5 | atcaagcggcccccttttttcac | ctcattgtcacactcctcgacagc |
| DUOX1 | tgtcaggctacgagatggtg | gttgctggacaggatgaggt |
| DUOX2 | tgacctggtgctgctgtttag | gcatcatggtgaagaattcgtc |
| PKC-δ | tgcctcaccgattcaaggtc | cttgccataggtcccgttgt |
| GAPDH | tggacattgttgccatcaacgacc | tggtgcaggatgcattgctgacaa |

## Supplementary Table 3. Primer sequence information for Q-PCR.

| Gene | Forward (5’->3’) | Reverse (5’->3’) |
|---|---|---|
| NOX1 | gagtgaaagtcatccccgca | taaaaagcaatcggcgcgag |
| NOX4 | gtctgcttgtttggctgtcc | acacaatcctaggcccaaca |
| GAPDH | gtcagtggtggacctgacct | tgagcttgacaaagtggtcg |

| Gene | Forward (5’->3’) | Reverse (5’->3’) |
|---|---|---|
| $p22^{phox}$ | cctccacttactgctgtccg | tggtaggtggctgcttgatg |
| $P47^{phox}$ | ggtgagatccacacagaaaaca | ggaggtgagggatgactctgt |
| $P67^{phox}$ | gctggcagcggacaagaaggact | ggcacaaacccaaatagcacacg |
| NOXO1 | gactgagggcaggactgttc | cttgggtccgttgttcctgt |
| NOXA1 | catccctgatgaccacaact | agtccaagtcctccggcctt |
| GAPDH | aggtcggtgtgaacggattt | ccactttgtcacaagagaaggc |
